# Increasing the attractiveness of surgical disciplines for students: Implications of a robot-assisted hands-on training course for medical education

**DOI:** 10.3389/fsurg.2022.953565

**Published:** 2022-07-21

**Authors:** Jonas Ekrutt, Sami-Ramzi Leyh-Bannurah, Sophie Knipper, Frederik Schramm, Burkhard Beyer, Tobias Maurer, Markus Graefen, Lars Budäus

**Affiliations:** ^1^ Martini-Klinik Prostate Cancer Center, University Hospital Hamburg-Eppendorf, Hamburg, Germany; ^2^ Department of Urology, St. Antonius-Hospital Gronau GmbH, Gronau, Germany; ^3^ Department of Urology, Asklepios Klinik Wandsbek, Hamburg, Germany; ^4^ Department of Urology, University Hospital Hamburg-Eppendorf, Hamburg, Germany

**Keywords:** evaluation, mentoring, curriculum, simulation, robotic surgery

## Abstract

**Background:**

Structured implementation of robot-assisted surgery in the field of medical education is lacking. We assessed students' interest in robot-assisted surgery and tested if the implementation of a hands-on robotic course into the curriculum could increase the interest to join a surgical discipline in general and especially in female students, since women are clearly underrepresented in surgical disciplines.

**Methods:**

After a prostate cancer focused seminar, 100 students were 1:1 randomized into two groups. Group B: Baseline characteristics and professional interest were assessed prior and after a hands-on robotic course, using a da Vinci® console with simulator (da Vinci® Surgical training, Intuitive Surgical Inc., USA). Group A served as post-interventional consistency control group, received the questionnaire only once after the hands-on training.

**Results:**

The male to female ratio of students was 54% and 46%. The interest to turn into urology/surgery, categorized as yes”, “no”, “maybe” changed from 18 to 16%, 36 to 30% and 46 to 54% respectively after the hands-on robotic course (*p* < 0.001). Also, the positive attitude towards the surgical field significantly increased (20 vs. 48%; *p* < 0.001). Comparing male and female students, virtually identical proportions (23 vs. 23%) opted for joining urology or surgery as a discipline, whereas rejection (45 vs. 25%) and perchance (32 vs. 50%) of that notion differed between genders (*p* = 0.12).

**Conclusion:**

Our results demonstrate great demand for implementing robotic training into medical education for an up-to-date curriculum. Although the decision process on career choice is widely multifactorial, stereotypes associated with surgical disciplines should be eliminated. This could have a particularly positive effect on the recruitment of female medical students since women are clearly underrepresented in surgical disciplines although currently and with increasing proportions, more female students are enrolled in medical schools then male.

## Introduction

Robotic assisted surgery has a firmly established role in the field of surgical disciplines resulting in an increased number of robotic assisted procedures and numerous international societies for robotic surgery. Nevertheless, a structured implementation in the field of medical education is lacking. Taking Germany as an example in 2019, 149 DaVinci® surgical systems were in use (source: Intuitive Surgical) whereas only one of 38 university hospitals did not offer robotic surgery. When asked by a letter-based survey the deaneries of 20 medical schools answered that they did not offer robotic surgery as an inherent part of the surgical curriculum for medical students. Although some medical schools offered it as part of block internships.

Currently, and with increasing proportions, more female students are enrolled in medical schools then male. Specifically, in the winter term 2018/2019, 62% (59,636 of 96,155) of enrolled medical students in Germany were female (62%) (1). Nonetheless women are still under-represented in the surgical profession in general. For example, in 2019 38,766 professional surgeons and 6,234 professional Urologists were registered in Germany of whom 8,419 (21.7%) and 1,196 (19.2%) were female (2). Similarly, in the UK and Australasia women accounted for just 12.9% of consultant (specialist) surgeons in 2019 (3).

Since robotic assisted surgery offers unique features (such as ergonomic working position, less physical effort, unlimited training opportunities *via* simulation etc.) we aimed at assessing the baseline interest of medical students in robotic assisted surgery and its curriculum implementation by a hands-on robot course. Our hypothesis stated that the features of robotic surgery could increase students' interest to join surgical disciplines in general and specifically in female students. Such a finding would allow us to secure the best talents for surgical disciplines and overcome potentially existing traditional stereotypes in surgical disciplines.

## Methods

### Study population

**Figure 1:**
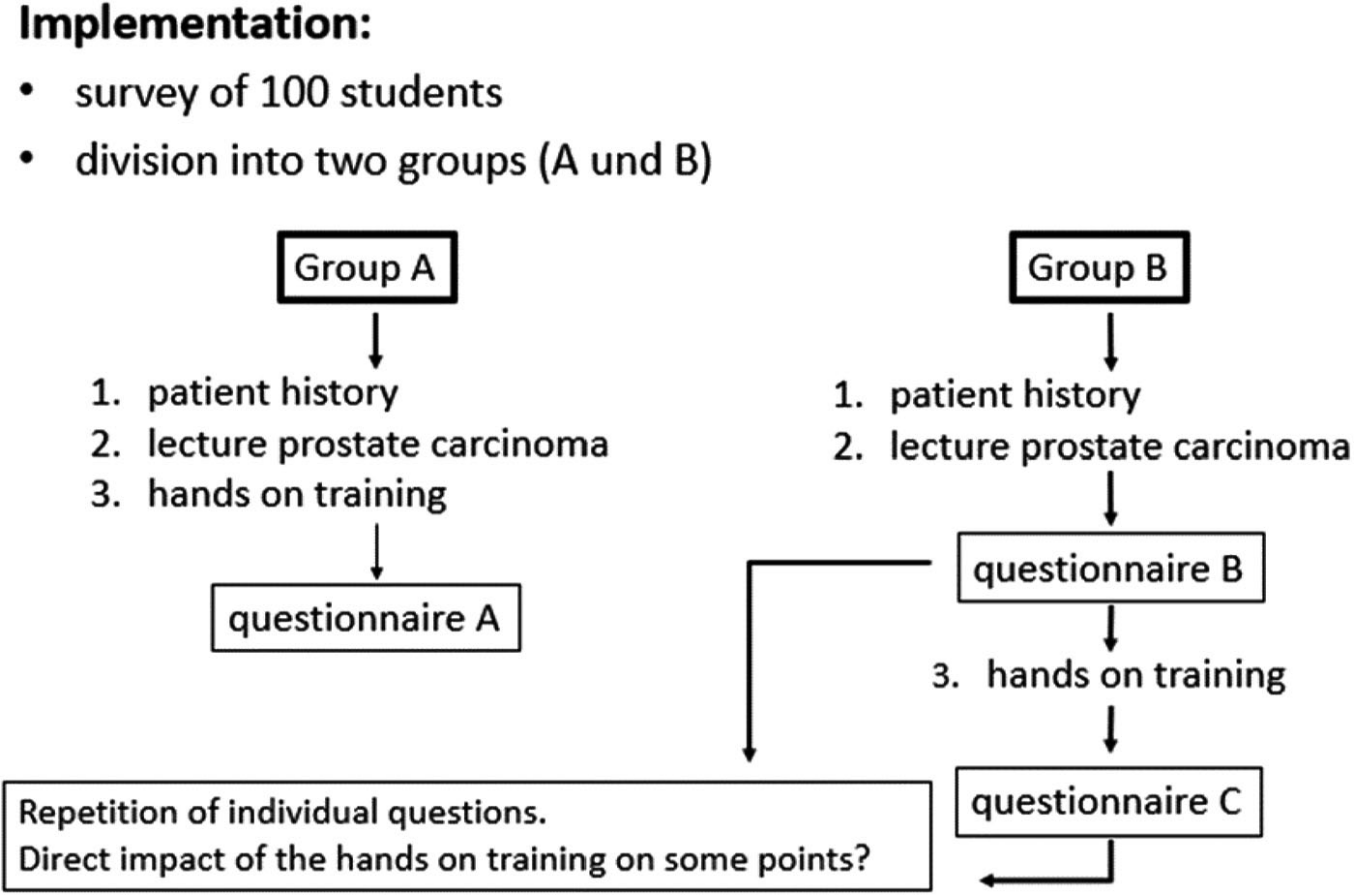
Study design.

After a general lecture on prostate cancer as part of a block internship, a total of 100 students were 1:1 randomized into two groups. Group B received a questionnaire in which, in addition to general questions such as age and current level of education, more specific questions such as the personal level of knowledge about robot-assisted operations, the interest in new technologies and the interest in a general implementation of robot-assisted training in student classes were assessed prior a hands-on robotic simulation course (da Vinci® Surgical training, “Pick & Place” and “Peg Board 1”, Intuitive Surgical Inc., USA). After the structured hands-on training, questions addressing the attractiveness of surgical disciplines were asked for a second time. Group A served as post-interventional consistency control group and received the questionnaire only once after the hands-on training (Figure 1).

### Statistical analyses

Descriptive statistics included frequencies and proportions for categorical variables. The Chi-square tested the statistical significance in proportions differences. For all statistical analyses R software environment for statistical computing and graphics (version 3.4.3) was used. All tests were two sided with a level of significance set at *p* < 0.05.

## Results

A total of 100 students, 56 of whom were female and 44 males, participated in the study. The average age was 25, ranging from 18 to 35 years. Most students attended the block internship during their 9 semesters of the regular study period (4–11 semester). 44 of students already finished a practical term in a surgical discipline and 84 students had already assisted during an operation. Of those, 10 took part in a robotic assisted surgery.

24 students had a medical parental background with parents of 8 students working in surgical disciplines. 84 students already had knowledge of robotic surgery before the block internship, especially though the internet and television as well as medical journals (Table 1).

**Table 1:** Demographic characteristics of all 100 participating students.

Variable	Group A (*n* = 50), *n* (%)	Group B (*n* = 50), *n* (%)	Overall (%)
Gender	f	28 (56%)	28 (56%)	56
	m	22 (44%)	22 ((44%)	44
Age	18–21	1 (2%)	6 (12%)	7
	22–25	35 (70%)	33 (66%)	68
	26–29	6 (12%)	8 (16%)	14
	30–34	7 (14%)	3 (6%)	10
	>35	1 (2%)	0 (0%)	1
Physicians Child	yes	11 (22%)	13 (26%)	24
	no	37 (74%)	37 (74%)	74
Surgeons Child	yes	2 (4%)	5 (10%)	7
	no	9 (18%)	8 (16%)	17
Surgical discipline as preferred carrer (especially urology)	yes	15 (30%)	9 (18%)	24
	no	21 (42%)	18 (36%)	39
	maybe	14 (28%)	23 (46%)	37
Surgical experience	yes	39 (78%)	45 (90%)	84
	no	11 (22%)	5 (10%)	16
Knowlege of robotic surgery	yes	40 (80%)	44 (88%)	84
	no	10 (20%)	6 (12%)	16

Overall, 96 students stated that they experienced the block internship as an enrichment of their curriculum and 77 of the students quoted that they would choose robot assisted surgery on themselves.

82 students stated that they would like to have the opportunity to join a mentoring programme in robotic surgery consisting of continuous meetings, internships during the semester break, insights into everyday clinical practice and regular robotic training sessions.

82 students (including 44 female students) were of the opinion, that robotic operating systems could make surgical disciplines more attractive as a workplace.

When asked specifically, in the whole cohort as well as among the female students, the main reasons for this were better and repetitive training options (simulation) (*n* = 71; *n* = 41), increased ergonomic working posture (*n* = 65; *n* = 35) and minimal invasiveness (*n* = 52; *n* = 30).

In response to the question whether the attributes of robotic operating systems could make surgical disciplines more attractive for women to choose as a career, 56 Students of whom 33 (58.9%) were female answered yes.

In group B the interest to turn into urology/surgery, categorized as yes”, “no”, “maybe” in changed from 18 to 16%, 36 to 30% and 46 to 54% respectively after the hands-on RARP course (*p* < 0.001) (Table 2). Furthermore, the positive perception regarding surgical disciplines in group B increased significantly after completion of the training (20 vs. 48%; *p* < 0.001) (Table 3).

**Table 2:** Group B: Do you want to work in a surgical field (especially urology)? (*p* < 0.001).

Answer	Before training, *n* (%)	After training, *n* (%)	*p* (*χ*^2^)
Yes	9 (18)	8 (16)	<0.001
No	18 (36)	15 (30)	
Maybe	23 (43)	27 (54)	

**Table 3:** Group B: positive attitude towards a surgical discipline before and after robotic hands-on training (*p* < 0.001).

	Before training, *n* (%)	After training, *n* (%)	*p* (*χ*^2^)
Yes	10 (20)	24 (48)	<0.001
Unchanged	31 (62)	22 (44)	
No	6 (12)	2 (4)	

## Discussion

Currently, medical disciplines are significantly influenced by technology. The same applies to surgery, where technology and digitalization are supposed to make processes faster, safer, and more efficient for the benefit of patients and healthcare workers. As part of this development, robotic operating systems are already an integral part of many operating theatres worldwide with robotic surgery being the gold standard for some indications (4). Since the numbers of robotic surgeries are constantly on the rise it seems beneficial that medical students, regardless of their later career choice, are familiar with robotic operating systems and the way procedures are performed with robotic support because they are very likely be caring for patients who have undergone or will undergo robotic surgery at some point. Additionally, profound knowledge of these techniques enables all future practitioners to better inform patients about the advantages and disadvantages of robotic surgery (5).

Before the block internship most students (*n* = 84) already knew that robotic surgery exists with information originating mainly from the internet and medical journals. This demonstrates the presence of robotic surgery in the media, as one potential reason for the increasing number of procedures performed (6, 7).

An important factor potentially arousing the interest of medical students in surgical disciplines is the early practical involvement in the subject. Although the decision process is widely multifactorial, a study by Santini et al. demonstrated that a surgeon-led clinical anatomy course in combination with clinical clerkships can result in a higher-than-average surgery matriculation rate (8).

Furthermore, the active involvement in surgical procedures is perceived positively, which in turn can positively influence the decision to start a surgical career (9).

In addition to the active involvement in the surgical procedure, a friendly environment and role models in the surgical profession are considered positive factors on career aspirations by the students (9). If medical students only take on the role of observers without active involvement in robotic procedures, they perceive robotic surgery as rather demotivating (10).

At the same time, it is important to question and, if necessary, eliminate stereotypes that might play a role in the selection of a medical discipline to attract the best talents for the respective discipline. Hill et al. described that the stereotypes and perceptions of surgery (competitive, masculine, and requiring sacrifice) and surgeons (self-confident and intimidating) can limit students' interest in surgery (11). Especially the perceived surgical personality and surgical culture seems to be a sex-specific deterrence to a career in surgery for women (12). This is an important finding, especially against the background of the increasing number of female students and the simultaneous underrepresentation of women in the surgical disciplines. For example, in our study population the gender ratio was 56 women to 44 men which reflects well the current general gender ratio among medical students in Germany (1).

By breaking down these stereotypes, surgical disciplines could be made more attractive to female students. This in turn could lead to a higher number of female surgeons who could serve as role models for female students.

A study by Bettis et al. showed: “it was not critical to be mentored by a woman to have a successful surgical career, but it was important to see a female surgeon role model, to see that surgical careers for women are real and attainable. Simply seeing a female in a surgeon's role was extremely influential and empowering” (13).

Despite its strength, the study also has its limitations. As already described, the decision-making process towards a particular specialty is multifactorial. All factors involved in this process are subjectively evaluated quite differently and cannot be objectively represented.

Accordingly, it will not be possible to create the teaching experience for all students through surveys and practical training. Specifically, the teaching methods generally perceived as enriching, such as active involvement in procedures, training opportunities on robotic operating systems, eliminating stereotypes, mentoring programs, etc., should be considered in the development of an up-to-date curriculum, in order to inspire young colleagues to start a career in a surgical discipline.

## Conclusions

In conclusion, our study contributes to the understanding of what medical students expect of a modern surgical education. The results demonstrated great demand for implementing robotic training into medical education. Overall, 82% of the students were interested in joining a mentoring program in robotic surgery. Additionally, 82% (including 78.6% of the female students) had the opinion, that robotic operating systems could make surgical disciplines more attractive as a workplace. By taking these findings into account, the attractiveness of surgical disciplines could be increased, which in turn could encourage more medical students to pursue a career in urology and surgical disciplines in general. Even if the training does not lead to a surgical career, it serves to broaden the medical horizon and could thus contribute to a better treatment and information about treatment options for patients (5).

Moreover, our findings indicate the great importance of eliminating stereotypes, especially to attract more female students since women are clearly underrepresented in surgical disciplines although, and with increasing proportions, more female students are enrolled in medical schools than male (1).

## Data Availability

The raw data supporting the conclusions of this article will be made available by the authors, without undue reservation.
